# Rates of inappropriate antiretroviral prescription among injection drug users

**DOI:** 10.1186/1477-7517-4-2

**Published:** 2007-01-04

**Authors:** Evan Wood, Robert S Hogg, Thomas Kerr, Simon Bonner, Steffanie A Strathdee, Anita Palepu, Julio SG Montaner

**Affiliations:** 1 British Columbia Centre for Excellence in HIV/AIDS, St. Paul's Hospital, 608 – 1081 Burrard Street, Vancouver, BC, V6Z 1Y6, Canada; 2 Department of Medicine, Faculty of Medicine, University of British Columbia, 3300 – 950 West 10th Avenue, Vancouver, BC, V5Z 4E3, Canada; 3 Faculty of Health Sciences, Simon Fraser University, 8888 University Drive, Burnaby, BC, V5A 1S6, Canada; 4 Department of Family and Preventive Medicine, University of California, San Diego, 9500 Gilman Dr., La Jolla, CA 92093, USA

## Abstract

**Background:**

Although the survival benefits of antiretroviral therapy (ART) for the treatment of HIV infection are well established, the clinical management of HIV disease continues to present major challenges. There are particular concerns regarding access to appropriate HIV treatment among HIV-infected injection drug users (IDU).

**Methods:**

In a prospective cohort study of HIV-infected IDU in Vancouver, Canada, we examined initial ART regimens vis-à-vis the provincial government's therapeutic guidelines at the time ART was initiated. Briefly, there have been four sets of guidelines: Era 1 (1992 to November 1995; double-drug (dual NRTIs) ART for patients with a CD4 cell count of 350 or less); Era 2 (December 1995 to May 1996; double-drug therapy for patients with a CD4+ cell count of 500 or less); Era 3 (June 1996 to June 1997; triple-drug therapy (dual NRTIs with a PI or NNRTI) for patients who had a plasma viral load of > 100,000 HIV-1 RNA copies/mL; dual therapy with two NRTIs for those with a plasma viral load of 5,000 to 100,000 HIV-1 RNA copies/mL); Era 4 (since July 1997; universal use of triple drug therapy as first-line treatment).

**Results:**

Between May 1996 and May 2003, 431 HIV-infected individuals were enrolled into the cohort. By May 31, 2003, 291 (67.5%) individuals had initiated ART. We noted instances of inappropriate antiretroviral prescription in each guideline era, with 9 (53%) in Era 1, 3 (12%) in Era 2, 22 (28%) in Era 3, and 23 (15%) in Era 4. Of the 57 subjects who received an inappropriate ART regimen initially, 14 never received the appropriate therapy; among the remaining 43, the median time to the initiation of a guideline-appropriate ART regimen was 12 months (inter-quartile range 5 – 20).

**Conclusion:**

The present study identified measurable rates of guideline-inappropriate ART prescription for patients who were injection drug users. Rates were highest in the era of dual therapy, although high rates persisted into the triple-therapy era. As therapeutic guidelines continue to evolve, it is critical that mechanisms be put in place to ensure prescription of ART combinations for IDU that are consistent with current expert recommendations.

## Background

Since the introduction of antiretroviral therapy (ART) in the mid-1990s, the survival benefits of this treatment for the management of HIV infection have been well established [1-4]. Nevertheless, the clinical management of HIV disease continues to present major challenges. Persons undergoing treatment for HIV disease must follow a daily regimen, known as highly active antiretroviral therapy (HAART), consisting of at least three antiretroviral drugs (i.e., two nucleoside reverse transcriptase inhibitors [NRTIs] plus a protease inhibitor [PI] or a non-nucleoside reverse transcriptase inhibitor [NNRTI]), and follow a scheduled dosing protocol that often involves coordination of dietary intake [5]. Previous studies have demonstrated that persons initially prescribed non-HAART regimens consisting of only one or two antiretroviral drugs have a lesser virologic response [19, 20], as well as significantly shorter survival than persons who initiate therapy with HAART [21, 22].

Since the advent of ART, there have been growing concerns regarding access to HIV treatment among HIV-infected injection drug users (IDU) [6,7]. Studies have demonstrated that IDU may be less likely to receive ART, even in settings where all HIV/AIDS care is provided free of charge [8]. We have recently shown that in comparison to non-injection drug users, IDU are more likely to be prescribed non-HAART regimens, even after adjustment for baseline clinical characteristics [9]. However, a comprehensive examination of the prevalence of inappropriate ART prescription among injection drug users has not previously been conducted. We therefore examined the rate of ART in a prospective cohort of HIV-infected injection drug users and examined the prevalence of ART prescriptions that were inappropriate, given the recommendations of therapeutic guidelines at the time ART was initiated.

## Methods

The Barriers to Antiretroviral Therapy (BART) cohort, a prospective study of HIV-infected injection drug users who have been recruited through self-referral and street outreach from the Downtown Eastside of Vancouver, Canada, since May 1996, has been described in detail previously [6,10]. Briefly, participants complete an interviewer-administered questionnaire, are provided referral to primary health care and addiction treatment where available, and are provided a nominal stipend at each study visit. Ethical approval has been annually provided by the University of British Columbia's Research Ethics Board. The BART cohort is unique in that it does not rely on self-reported use of ART, since endpoints related to the use of antiretroviral therapy can be accurately ascertained through a confidential record linkage with the province's centralized HIV/AIDS monitoring and treatment registry which includes all patients receiving ART in the province of British Columbia [6,10,11].

The primary endpoint of interest in the present analysis was the content of the initial ART regimen, and we were specifically interested in the rate of antiretroviral therapy use that was inconsistent with the recommendations of the province's therapeutic guidelines at the time antiretroviral therapy was initiated. The guidelines in the province of British Columbia have been described in detail previously [2,12]. Briefly, between 1992 and November 1995 (Era 1), the guidelines made available double-NRTI antiretroviral therapy for people with a CD4 cell count of 350 or less; between December 1995 and May 1996 (Era 2), double-NRTI therapy was made available to everyone with a CD4+ cell count of 500 or less; between June 1996 and June 1997 (Era 3), antiretroviral therapy guidelines based on plasma viral load were used, and individuals who had a plasma viral load of > 100,000 HIV-1 RNA copies/mL were offered triple-drug regimens (i.e., HAART), whereas those with a plasma viral load of 5,000 to 100,000 HIV-1 RNA copies/mL were offered dual NRTI therapy; and in July 1997 (Era 4), the guidelines universally recommended the use of HAART as first-line treatment.

## Results

Between May 1996 and May 2003, 431 HIV infected individuals were enrolled into the BART cohort. Antiretroviral use was subsequently prospectively and retrospectively examined for these individuals through a confidential record linkage with the province's centralized HIV/AIDS treatment program, and it was determined that 291 (67.5%) individuals had initiated antiretroviral therapy by May 31, 2003. Overall, 19 (6.9%) individuals had to be excluded from subsequent analyses because baseline plasma HIV RNA measures and/or baseline CD4 cell count measures were unavailable.

Among the study sample of 272 individuals, the median age of these participants was 36 (inter-quartile range: 30 – 42), 118 (43.4%) were female, and 115 (42.3%) reported being of non-white race. Overall, 17 (6.3%) participants initiated ART in Era 1, 26 (29.4%) in Era 2, 80 (29.4%) in Era 3, and 149 (54.8%) in Era 4.

We noted that there were instances of inappropriate antiretroviral prescription in each era, with 9 (53%) in Era 1, 3 (12%) in Era 2, 22 (28%) in Era 3, and 23 (15%) in Era 4. In Eras 1 and 2, all inappropriate prescriptions involved individuals being prescribed mono therapy when they should have received dual therapy based on their CD4 cell count. In Era 3, all inappropriate prescriptions involved subjects who should have received triple therapy based on their plasma HIV RNA but received dual therapy instead (2 NRTIs in all cases). Finally, in Era 4, when patients should have received triple therapy, 4 patients received single NNRTI therapy, 3 patients received single NRTI therapy, 6 patients received dual therapy with one NRTI and a PI or a NNRTI, and 10 patients received dual NRTI therapy. Overall, of the 57 subjects who received an incorrect ART prescription initially, 14 never received the correct number of drugs, and among the remaining 43, the median time to the initiation of a correct ART regimen was 12 months (inter-quartile range 5 – 20). Interestingly, when we compared those to received appropriate ART to those that received guideline-inappropriate ART, we found that no physician or patient characteristics were associated with receiving inappropriate therapy.

## Discussion

In the present study, we documented measurable rates of ART prescription that were inconsistent with the recommendations of the therapeutic guidelines that were in place at the time that ART was initiated. Our findings may be explained by the difficulty some physicians may have had in keeping up with changes in HIV therapeutic guidelines and by the fact that inappropriate prescriptions commonly resulted from lack of knowledge about the change in guidelines [9]. In addition, limited access to appropriate ART combinations among persons with a history of injection drug use may reflect the belief of some physicians that persons with less stable lifestyles may have better adherence to a less complex regimen [7,13]. Finally, it is also possible that concerns regarding possible transmission of PI- or NNRTI-resistant virus influenced prescribing decisions [14,15].

With regard to the above, it is noteworthy that a recent study demonstrated that a substantial proportion of homeless and marginally housed individuals had good adherence to antiretroviral therapy including protease inhibitors, and that resistance to PIs was rare among those who were non-adherent [25]. Furthermore, it was recently argued that physicians should not indefinitely withhold ART from patients who are thought to be poorly adherent [26], and studies have shown that providers may be poor judges of adherence [27]. The present study demonstrates the high rate of inappropriate ART prescription but is limited in its ability to explain why measurable numbers of IDU received incorrect HAART regimens. Future studies should examine physician and patient reasons for selecting specific ART regimens. Studies are also needed to examine ways to ensure physician compliance with therapeutic guidelines since it is likely that HIV therapies will continue to be multi-drug, and that therapeutic guidelines will continue to evolve rapidly [16]. In our own setting where HAART delivery is centralized, systems are now in place to ensure all prescriptions are consistent with current therapeutic guidelines.

## Conclusion

In the present study, we documented measurable rates of inappropriate ART prescribing patterns among injection drug users. Rates were highest in the era of dual therapy, although high rates persisted into the era of HAART. Since HIV therapeutic guidelines will likely continue to evolve as novel agents become available and additional information about ART benefits and toxicities arises, it is critical that mechanisms be put in place to ensure that physicians are providing ART combinations that are up-to-date with current knowledge.
